# The role of noncoding RNAs in epithelial cancer

**DOI:** 10.1038/s41420-020-0247-6

**Published:** 2020-03-12

**Authors:** Massimiliano Agostini, Carlo Ganini, Eleonora Candi, Gerry Melino

**Affiliations:** 1 Department of Experimental Medicine, TOR, University of Rome “Tor Vergata”, 00133 Rome, Italy; 2 IDI-IRCCS, Via Monti di Creta 106, 00166 Rome, Italy; 3 MRC Toxicology Unit, University of Cambridge, Department of Pathology, Tennis Court Road, Cambridge, CB2 1QP UK

**Keywords:** Long non-coding RNAs, miRNAs

## Abstract

Regulatory noncoding RNAs (ncRNAs) are a class of RNAs transcribed by regions of the human genome that do not encode for proteins. The three main members of this class, named microRNA, long noncoding RNA, and circular RNA play a key role in the regulation of gene expression, eventually shaping critical cellular processes. Compelling experimental evidence shows that ncRNAs function either as tumor suppressors or oncogenes by participating in the regulation of one or several cancer hallmarks, including evading cell death, and their expression is frequently deregulated during cancer onset, progression, and dissemination. More recently, preclinical and clinical studies indicate that ncRNAs are potential biomarkers for monitoring cancer progression, relapse, and response to cancer therapy. Here, we will discuss the role of noncoding RNAs in regulating cancer cell death, focusing on those ncRNAs with a potential clinical relevance.

## Facts

- Many of the genetic and epigenetics alterations in human cancers are found in noncoding regions of the DNA.
- miRNAs and lncRNAs have been largely described in their role in controlling epithelial tissues homeostasis, both in physiological and pathological conditions such as cancer.
- The clinical application of ncRNAs is already under evaluation in ongoing clinical trials exploring their role as biomarkers for patient survival, metastasis development prediction, or therapy response.

## Open questions

- What is the role of regulatory ncRNA in pyroptosis, ferroptosis, and autophagy?
- What is the functional interplay between miRNA, lncRNA, and circRNA in cancer biology?
- How can we integrate preclinical findings to select the best regulatory ncRNA for clinical studies?

## Introduction

Protein-coding genes undoubtfully play an established role in cancer transformation and progression, however, recent compelling evidence is highlighting a role for noncoding RNAs. There is no question that loss or mutation of crucial genes are frequent events in cancer biology: the mutation of the tumor suppressor gene p53 is observed in almost 50% of human cancers^1–7^, and the amplification of the oncogene MYC^8^ or the translocation of BCL-2 gene^9–12^ are crucial drivers in various cancer contexts^13–16^. However, many of the recurrent genetic and epigenetic alterations are found in genes that do not codify for proteins but that codify for an entire class of molecules called noncoding RNAs (ncRNAs), which play a key role in the regulation of many cellular activities.

The non-protein-coding regions of the human genome are transcribed into molecules of RNA classified as regulatory noncoding RNAs^17^. ncRNAs are mainly transcribed by RNA polymerase II and share several characteristics with messenger RNAs (mRNAs). Indeed, they have a cap structure at the 5′ end, a poly(A) tail at the 3′ end and their expression is controlled by canonical promoter elements and transcription factors. Conventionally, regulatory ncRNAs are classified either as small ncRNAs, if they are shorter than 200 ribonucleotides or as long noncoding RNAs (lncRNAs), longer than 200 ribonucleotides. Small ncRNAs include microRNAs (miRNAs), which mediates post-transcriptional RNA silencing, piwiRNAs, which regulate chromatin modifications and transposons repression, as well as the more recent circular RNAs (circRNAs)^18^.

In this review, we shall discuss the role of ncRNAs in regulating cancer cell death (Table 1)^19^. In particular, we will describe those regulatory ncRNAs that have been largely investigated, on the basis of both in vitro and in vivo evidence as clinical data.

**Table 1:** Pro-apoptotic and anti-apoptotic ncRNAs.

ncRNA	PRO-apoptotic role	Cellular/animal model	Relevant references (original papers)
Let-7	• Inhibition of BCL2L1 • Upregulation of BAK and BAX, and downregulation of BCL-xL	• Colorectal cancer • Leukemia	Mizuno et al.^46^ Huang et al.^47^
miR-15/16	• Repression of BCL-2 and BMI1	• Chronic lymphatic leukemia • Mantle cell lymphoma	Cimmino et al.^52^ Teshima et al.^53^
miR-34	• Regulation of proteins involved in cell death: BCL-2, BIRC5 (Survivin), CREB, and YY1	• Pancreatic cancer • Myeloid leukemia • Neuroblastoma • Laryngeal squamous cell cancer	Chang et al.^67^ Pigazzi et al.^68^ Chen et al.^69^ Shen et al.^70^
miR-29	• Repression of MCL-1 expression	• Acute myeloid leukemia	Garzon et al.^76^
GAS5	• Induction of apoptosis in a mouse model	• Brest cancer (mouse)	Mourtada-Maarabouni et al.^103^
MEG3	• Induction of p53	• Colorectal cancer	Zhou et al.^108^
NIKLA	• Activation of apoptosis in tumor-specific CTLs	• Breast cancer and lung cancer	Huang et al.^113^
NEAT1^a^	• Enhanced apoptosis after DNA damage	• Chronic lymphocytic leukemia	Blume et al.^116^
circFOXO3	• Increased PUMA expression	• Breast cancer (mouse)	Du et al.^147^

**ncRNA**	**ANTI-apoptotic role**	**Cellular/animal model**	**Relevant references (original papers)**
miR-21	• expression of pro-apoptotic genes: APAF11, PDCD4, RHOB, and FASLG	• Non-small cell lung cancer	Hatley et al.^84^
miR-155	• Repression of SHIP-1	• B lymphocytes • Hematopoietic cells	Costinean et al.^91^ O’Connell et al.^92^
miR-221	• Increased apoptosis and cell-cycle arrest	• Hepatocellular carcinoma	Park et al.^98^
CCAT	• Interaction with p63 and SOX2	• Squamous cell carcinoma	Jiang et al.^123^
FAL1	• Interaction with BMI1	• Pan-cancer database	Hu et al.^128^
PVT1	• Downregulation of Caspase-7, Caspase-9, and PARP	• Nasopharyngeal carcinoma	He et al.^132^

^a^Controversial role since NEAT1 has also been correlated to an oncogenic activity in some tumor types.

### Epithelial ncRNAs

Recently, miRNAs and lncRNAs have gained significant attention for their role in controlling epithelial tissue homeostasis in normal and pathological conditions, being key regulators of epithelial progenitor stem cells proliferation, somatic lineage specification, and differentiation^20,21^.

Distinct miRNAs control epidermal development, epidermal differentiation, and adult stem cells maintenance (for reviews see refs. ^22,23^). These include the miR-20 family, miR-24, the miR-200 family, miR-205, and miR-203^24–28^. The latter has been described in his mechanistic role in the control of epithelial progenitor cells proliferation and in the inhibition of cellular senescence, having as a target one of the epithelial master genes, the transcription factor TP63^24,29^. Interestingly, these epithelial-specific miRNAs have also been implicated in controlling key regulators genes of the pathogenesis of many adenocarcinomas and squamous cell carcinomas (SCC)^30^. As an example, the miR-200 family, controlling the expression of ZEB1 and ZEB2, have been described in the epithelial-to-mesenchymal transition (EMT) in ovarian cancer and SCCs. Moreover, miR-203, targeting ABL1, SOCS3, and ZEB3, controls EMT in epithelial cancers^28,31^. miRNAs also play a key role in epithelial cells upon different environmental stressors, such us UV radiations and inflammation^32–36^.

Only a few lncRNAs have been studied so far in the regulation of epithelial development, such as ANCR, an anti-differentiation lncRNA, and TINCR, a terminal differentiation-induced lncRNA; however, the mechanism through which they maintain the cell of the epidermis in a undifferentiated state has not been identified yet^37,38^. Similarly, LIN00941 has also been implicated in the control of epidermal homeostasis repressing the expression of pro-differentiation genes through a not-yet identified mechanism^39^. Moreover, the pro-differentiation lncRNA, uc.291, has been shown acting as a pro-differentiation transcript by facilitating the activation/binding of the chromatin remodeling complex SWI/SNF (BAF) in proximity to the epidermal differentiation complex (EDC) genes to allow their expression^40^.

While several studies investigated the specific contributions of miRNAs in epithelial cancer development, the role of lncRNAs in this context has not been investigated thoroughly yet.

### microRNAs with pro-apoptotic functions

#### let-7

let-7 has been one of the first miRNAs isolated and characterized in the nematode *C. elegans*. The human genome contains 13 let-7 family member genes, which are distributed in different genomic loci and codify for 9 mature miRNAs^41^. Let-7 expression is downregulated in many cancers, such as lung, breast, pancreatic cancer, and melanoma and in many cancer-associated clinical conditions like cholestasis^42^. Moreover, downregulation of let-7 expression correlates with poor survival in lung cancer^43^, ovarian cancer^44^, and head and neck SCC patients^45^. Functionally, let-7 acts as a tumor suppressor partially through the downregulation of several genes involved in cell death, as shown by the ectopic expression of let-7 resulting in the inhibition of BCL2L1 in colorectal cancer^46^ or by the induction of apoptosis via upregulation of BAK and BAX and downregulation of BCL-XL protein levels^47^. Although in vivo delivery of let-7 in mouse models of lung cancer demonstrated its therapeutic potential, its tumor suppressor functions might be associated with repression of cell proliferation and elimination of cancer cells mainly through a non-apoptotic mechanism^48^.

#### miR-15/16

**Fig. 1:**
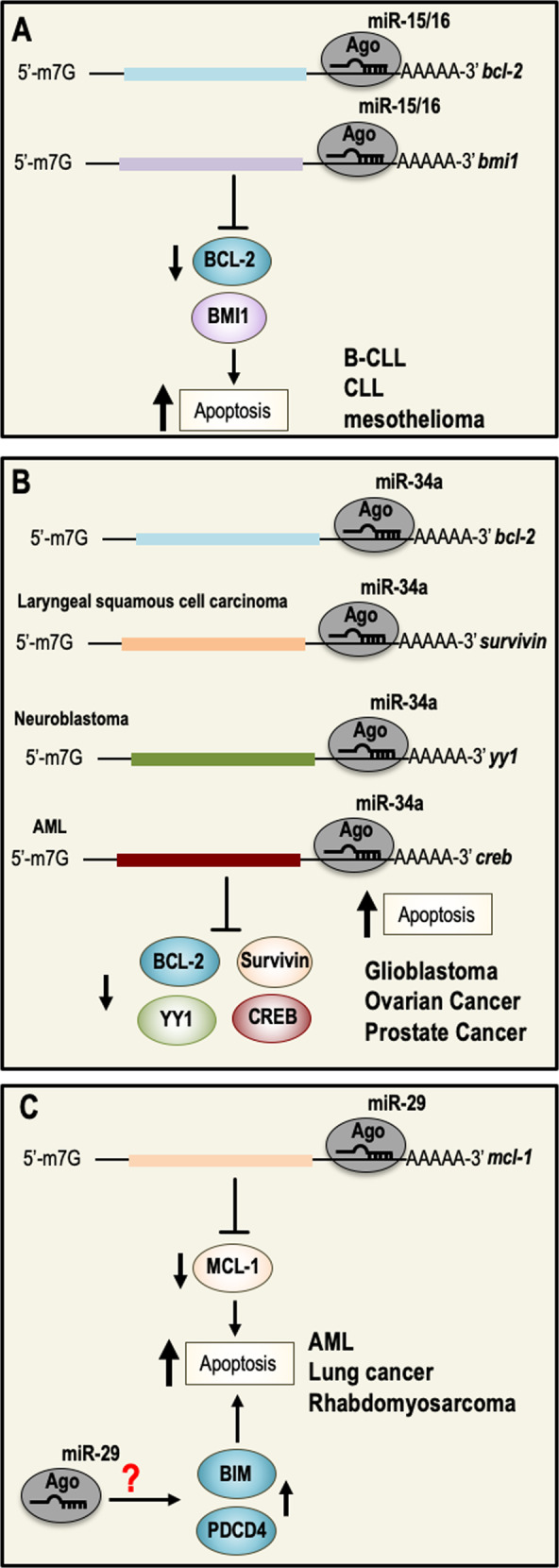
microRNAs with pro-apoptotic functions and relative inhibited molecular pathways. **a** miR-15/16 expression is deregulated in hematological malignancies and induces cell death by inhibiting the expression of the anti-apoptotic protein BCL-2 and BMI1. **b** miR-34a acts as a tumor suppressor gene by directly repressing the expression of several proteins involved in the regulation of cell survival. **c** miR-29a regulates the critical anti-apoptotic genes MCL-1. MCL-1 is an anti-apoptotic protein that promotes cancer cell survival and proliferation, and it is frequently overexpressed in AML. It has been also shown that miR-29 can induce cell death by inducing the expression of pro-apoptotic proteins, including BIM and PDCD4. BCL-2 B-cell lymphoma 2 gene, BMI1 polycomb complex protein BMI1, MCL-1 induced myeloid leukemia cell differentiation protein, BIM BCL-2-like protein 11, PDCD4 programmed cell death protein 4, AML acute myeloid leukemia.

miR-15 and miR-16 are localized on chromosome 13q14. This chromosomic region is frequently deleted in B-cell chronic lymphocytic leukemia (B-CLL)^49^. miR-15 and miR-16 are found in a 30-kb region, which is lost in CLL, and both genes are either deleted or downregulated in most of CLL cases (~68%)^50^. However, several studies suggested that miR-15/16 could be also downregulated by additional mechanisms, such as defective DROSHA processing or epigenetic alterations^51^. The tumor suppressor action of miR-15/16 is the result of the induction of cell death through the repression of the anti-apoptotic proteins BCL-2 and BMI1 (Fig. 1a)^52–57^. More recently, the tumor suppressor function of miR-15/16 has also been observed in solid tumors such as mesothelioma^58^ or chondrosarcomas^59^, where its expression is suppressed in order to promote tumor neo-angiogenesis. Moreover, the miR-15/16 knockout mouse model supports the in vivo tumor suppressor activity of miR-15/16. Indeed, deletion of miR-15/16 gene results in B cells proliferation and in the development of lymphoid malignancies^60,61^.

#### miR-34

The miR-34 family consists of three different transcripts miR-34a, miR-34b, and miR-34c with high sequence homology.

In humans, the miR-34a gene is located on the chromosomal region 1p36.22, which is frequently deleted in many human cancers, including neuroblastoma, glioma, breast cancer, lung cancer, colorectal cancer, and melanoma^62–64^. The miR-34b and miR-34c genes are both encoded on chromosome 11q23.1, and rearrangements of this region have been observed in several solid tumors and in hematological malignancies^65^. Both in vitro and in vivo studies have shown that the miR-34 family genes (in particular miR-34a) act as tumor suppressors: when overexpressed they repress several oncogenes, resulting in an increased cancer cell death and in an inhibition of metastasis development. miR-34a has also been shown to play a role in the regulation of NF-κB in CD8 + T cells, promoting their cytotoxic activity^66^. In the context of cell death, miR-34 family acts on proteins such as BCL-2, BIRC5 (Survivin), CREB, and YY1, which are involved in apoptosis regulation (Fig. 1b)^67–71^.

Nevertheless, it should be noted that miR-34-deficient animals are not showing increased susceptibility to either spontaneous or to irradiation-induced nor MYC-initiated tumorigenesis.

Strikingly in 2013, a miR-34 mimic (MRX34) became the first microRNA tested in a phase 1 clinical trial (NCT002862145). In particular, a liposomal miR-34 mimetic was administered intravenously to patients with unresectable liver cancer or metastatic cancer refractory to standard treatment, with or without liver involvement. From these studies, MRX34 treatment was associated with antitumor activity and acceptable safety, but subsequent monitoring showed immune-related toxicities. To date, it is not clear whether further clinical trials will be started^72^.

#### miR-29

The miR-29 family is composed of three isoforms. miR-29b-1 and miR-29a form one cluster on chromosome 7q32, while miR-29b-2 and miR-29c form a second cluster on chromosome 1q23. Of note that the first cluster region is frequently deleted in myelodysplastic syndromes and therapy-related acute myeloid leukemia (AML)^73,74^. In addition, several experimental observations showed that miR-29 family is downregulated in chronic lymphocytic leukemia, lung cancer, invasive breast cancer, and cholangiocarcinoma. miR-29 functions as a tumor suppressor gene in several cancer types. Indeed, ectopic expression of miR-29b induced apoptosis in cholangiocarcinoma cell lines and reduced tumorigenicity in a xenograft model of lung cancer, rhabdomyosarcoma, and AML^75^. Mechanistically, miR-29 induces cell death by directly repressing the expression of the anti-apoptotic BCL-2 family member, MCL-1 (Fig. 1c)^76^. In addition, modulation of miR-29 expression results in the upregulation of some pro-apoptotic genes, such as BIM and the tumor suppressor programmed cell death-4 (PDCD4) in AML, and also inhibits the expression of DNMT3B in hepatocarcinoma cell lines^77^. Interestingly, MCL-1 mRNA inversely correlated with miR-29a or miR-29b expression in 45 primary AML samples^78^.

### microRNAs with anti-apoptotic functions

#### miR-21

**Fig. 2:**
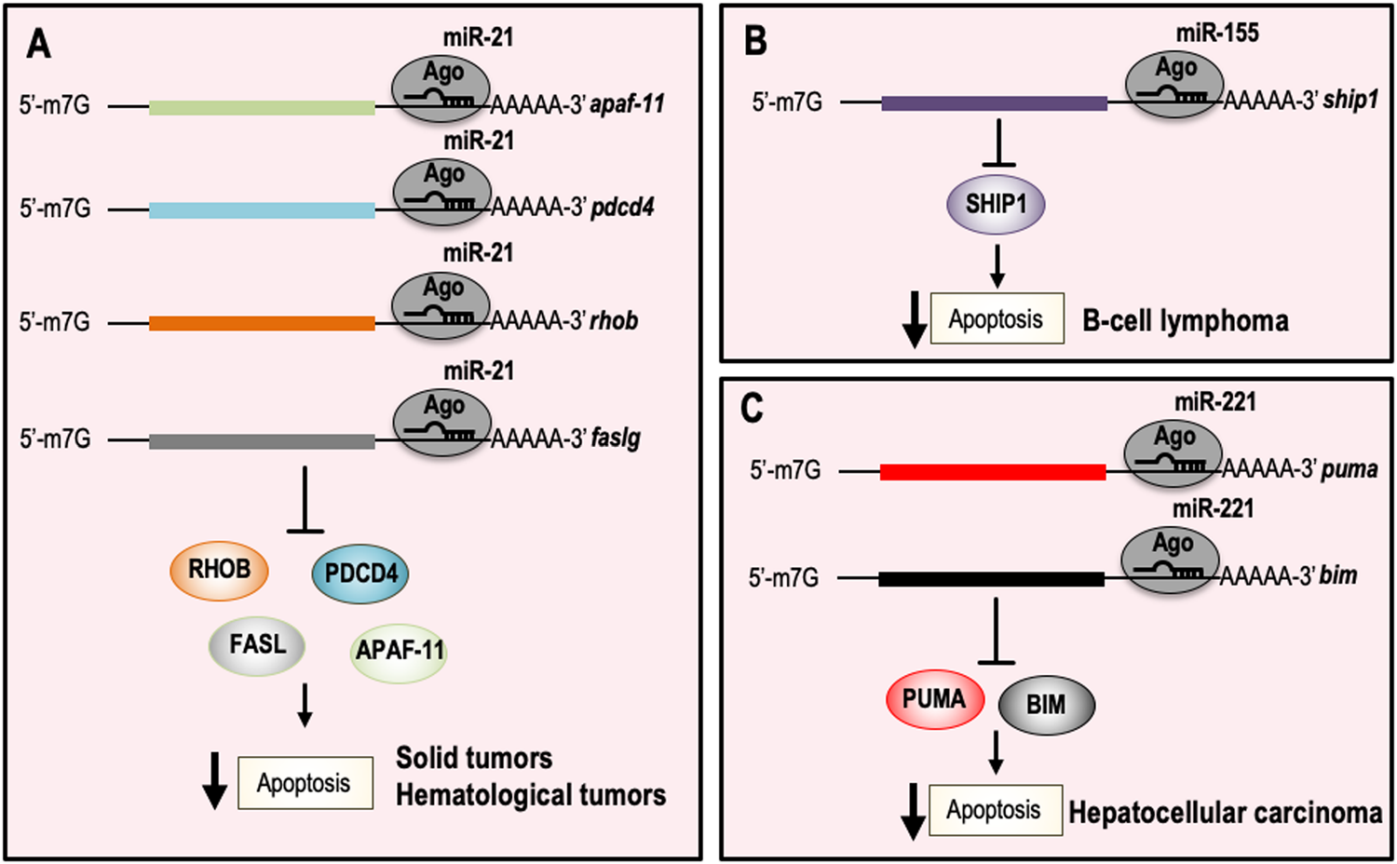
microRNAs with anti-apoptotic functions and relative inhibited molecular pathways. **a** miR-21 is the most upregulated oncomiR in solid tumors and hematological malignancies. The oncogenic activity of miR-21 is associated with the direct negative regulation of pro-apoptotic genes, such as APAF11, PDCD4, RHOB, and FASLG. **b** miR-155 plays a key role in the regulation of the immune response. miR-155 is upregulated in B-cell lymphomas and by targeting SHIP-1 supports cell survival. **c** In liver cancer, miR-221 exerts oncogenic activity by repressing the expression of the apoptotic proteins PUMA and BIM. APAF11 apoptotic protease activating factor 1, PDCD4 programmed cell death protein 4, RHOB Ras Homolog Family Member B, FASLG Fas ligand, SHIP-1 Src homology 2-SH2 domain containing inositol polyphosphate 5-phosphatase 1, PUMA p53 upregulated modulator of apoptosis, BIM BCL-2-like protein 11.

The human miR-21 gene is located in the fragile site FRA17B on chromosome 17q23.2^79^. Expression profile studies on tumor samples, including lung, breast, stomach, prostate, colon, pancreatic tumors, and B-cell lymphomas showed that miR-21 is the most commonly upregulated miRNA in solid tumors and hematological malignancies, indicating miR-21 as an “oncomiR”^80–82^. Indeed, functional studies in several cancer cell lines demonstrated that knockdown of miR-21 activates caspases leading to apoptotic cell death. Moreover, in vitro evidence also suggested a positive regulatory role in pyroptosis through the activation of the NLRP3 inflammasome^83^. At a molecular level, miR-21 suppresses the expression of pro-apoptotic genes, such as APAF11, PDCD4, RHOB, and FASLG (Fig. 2a)^84^. The oncogenic role of miR-21 was also confirmed in vivo by generating conditionally expressing miR-21 mice^84^. This mouse model demonstrated that miR-21 is capable of initiation, maintenance, and prolonged survival of tumors in vivo and demonstrated the importance of miR-21 in hematological malignancies^85^.

#### miR-155

The human gene miR-155 is localized on chromosome 21 within an exon of a noncoding RNA, from the B-cell Integration Cluster (BIC)^86^. miR-155 is mainly expressed in lymphoid tissues, including thymus and spleen, where it regulates several physiological functions of these organs, such as antibodies and cytokines production^87^. In addition, several observations have shown that miR-155 is accumulated in non-Hodgkin lymphomas, as in the diffuse large B-cell lymphomas, and Burkitt lymphomas and in Hodgkin lymphomas^88–90^. The oncogenic role of miR-155 was confirmed by the generation of genetically modified mice, developing B-cell lymphoma upon its overexpression. Among the molecular mechanisms underlying its oncogenic properties, the direct repression of SH2-containing inositol phosphatase (SHIP-1) (Fig. 2b)^91,92^, a positive regulator of apoptosis, seems to play a pivotal role. Apart from hematologic malignancies, miR-155 is also overexpressed in several solid tumors, such as breast, colon, pancreatic, and lung cancer, and has also been described in the regulation of autophagy in an experimental mouse model of pancreatitis^93^. More recently, the therapeutic efficacy of an anti-miR-155 was tested in vivo by using a novel delivery system targeting the acidic tumor microenvironment: a nucleic acid anti-miR-155 linked to a peptide with a low pH-induced transmembrane structure (pHLIP) effectively inhibited miR-155 expression after intravenous administration, leading to cancer regression in a mouse model of lymphoma^94^.

#### miR-221

miR-221/222 is a microRNA with oncogenic properties, highly conserved in vertebrates, located on the X chromosome in humans, mice, and rats. Overexpression of miR-221/222 has been observed in several human malignancies, including hepatocellular carcinoma, breast, prostate, pancreatic cancer, and glioblastoma^95,96^. The oncogenic activities of miR-221 were also confirmed in vivo as the overexpression of miR-221 in p53^−/−^; Myc liver progenitors stimulates tumor onset and progression^97^. The oncogenic activity of miR-221 might be partially explained by the repression of PUMA and BIM proteins (Fig. 2c). Interestingly, in vivo preclinical studies showed that a cholesterol-modified isoform of anti-miR-221 significantly reduced miR-221 levels in livers after injection, reducing tumor cell proliferation, and increasing the expression of apoptosis and cell-cycle arrest markers, eventually prolonging mice survival^98^. Although miR-221 is classified as an “oncomiR”, it should be noted that his role as tumor suppressor gene has also been observed^99^.

### Long noncoding RNAs

Long noncoding RNAs are classified as endogenous ncRNAs longer than 200 nucleotides. They are transcribed by RNA polymerase II from an independent promoter and processed as coding RNAs. Indeed, they are capped, spliced, and polyadenylated, however, lncRNAs lack a significant open-reading frame. Compared with protein-coding transcripts, lncRNAs are overall expressed at lower levels and they are not highly evolutionarily conserved, with only 5–6% of lncRNAs harboring conserved sequences^100^.

Several studies have described a range of molecular mechanisms by which lncRNAs may exert their functions. In particular, lncRNAs have been described as molecular scaffolds or architectural RNAs in a variety of cellular processes, among which epigenetics modifications, alternative splicing, mRNA translation, and maintenance, and acting as decoys or “sponges” for miRNAs or transcription factors.

### lncRNAs with pro-apoptotic function

#### GAS5

**Fig. 3:**
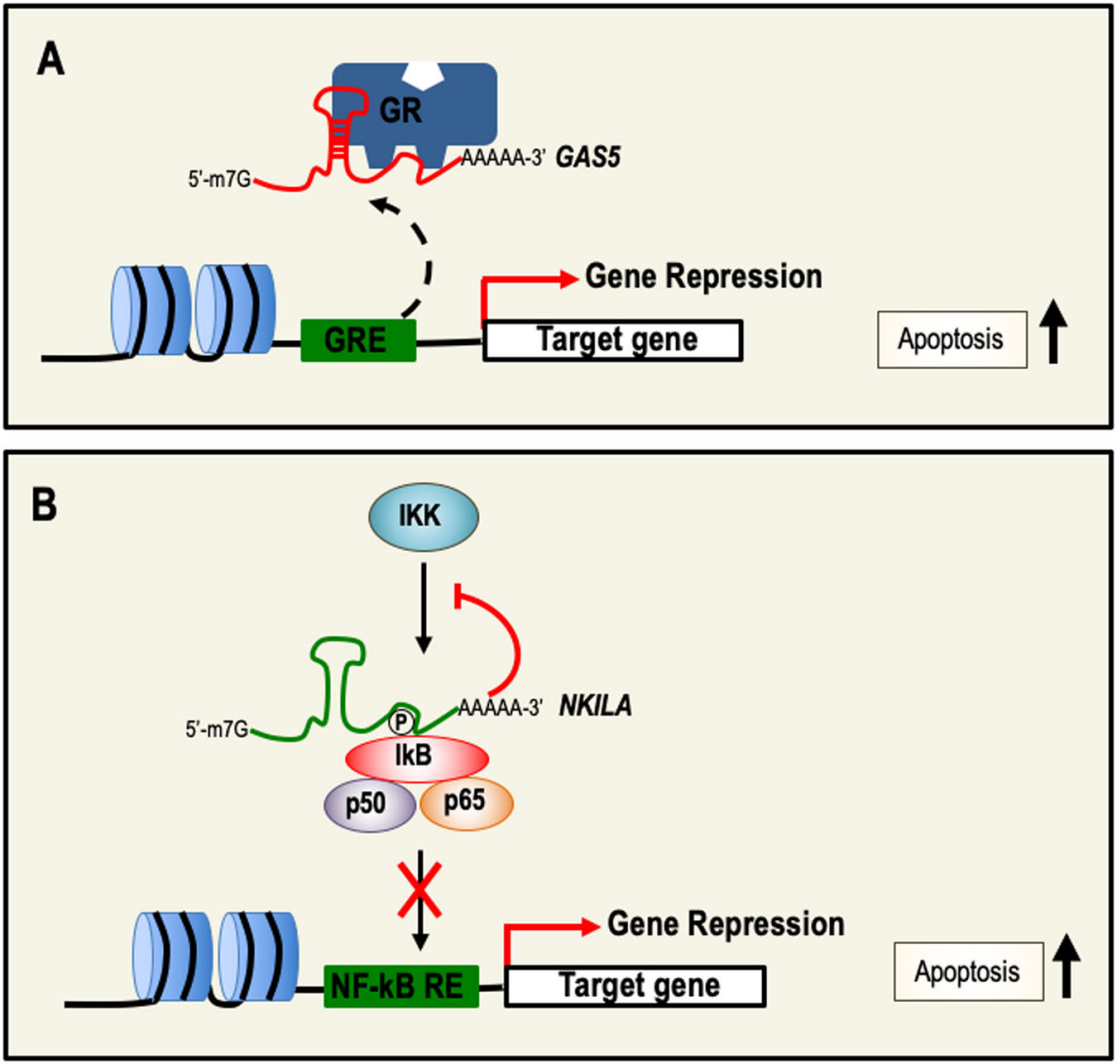
lncRNAs as inducers of cell death and relative molecular mechanisms. **a** GAS5 prevents GR-dependent gene activation by binding to the glucocorticoids receptor (GR) functioning as a decoy. **b** NF-κB is a key transcription factor in the regulation of cell survival. NKILA binds IkB and prevents its phosphorylation resulting in the inhibition of NF-κB activation, thereby repressing NF-κB target genes involved in cell survival. GAS5 growth arrest-specific transcript 5, GR glucocorticoids receptors, NF-κB nuclear factor kappa-light-chain-enhancer of activated B cells, IkB inhibitor of kappa B.

The growth arrest-specific transcript 5 (GAS5) is located at 1q25, and the gene transcribed contains 12 exons that do not encode for functional proteins^101^. GAS5 is a tumor suppressor gene, and is one of the most expressed lncRNAs in all human tissues^102^. The expression of GAS5 in cancers is significantly reduced. Reduced expression has been observed in breast, prostate, head and neck, gastric, colorectal, pancreatic, and cervical cancer, and more importantly, its expression is negatively correlated with clinical–pathological characteristics, such as tumor size, staging, or metastasis. Moreover, in vivo studies have shown inhibition of breast tumor growth by inducing cell-cycle arrest and apoptosis after the overexpression of GAS5 in breast cancer cell lines and their subsequent injection in nude mice^103^. Several molecular mechanisms of action for GAS5 have been proposed, including inhibition of translation and decoy or miRNA sponge activity. The decoy function of GAS5 has been shown in HeLa and HepG2 cell lines, and seems to be its major mechanism of action. Indeed, GAS5 interacts with the DNA-responsive elements of glucocorticoids receptor preventing the binding of the receptor to the DNA, thereby blocking the activation of target genes transcription (Fig. 3a)^104^. Moreover, GAS5 can also interact with other steroid hormone receptors including progesterone and androgen receptors, which play important roles in hormone-dependent cancers^102^.

#### MEG3

Maternally expressed gene 3 (MEG3) located on the human chromosome region 14q32.3 is expressed in many normal tissues^105^. However, MEG3 expression is lost in several human tumors including osteosarcoma, hepatocellular cancer, gastric cancer, and non-small cell lung cancer (NSCLC) by promoter or intergenic differentially methylated region hypermethylation, suggesting that loss of MEG3 expression contributes to tumor development in several tissues^106,107^. Importantly, patients with lower levels of MEG3 expression had a relatively poor prognosis. MEG3 functions as a tumor suppressor gene, and its re-expression inhibits cell proliferation and promotes apoptosis in human glioma and NSCLC cell lines. At a molecular level, MEG3 function is mediated, at least partially, by the activation of the tumor suppressor gene p53 by the downregulation of MDM2 expression^108–110^.

#### NKILA

The human gene that encodes NKILA, an NF-κB-interacting lncRNA, is located on chromosome 20. NKILA is downregulated in breast cancer, nasopharyngeal carcinoma, and melanoma^111^. In addition, reduced NKILA expression is associated with breast cancer metastasis and poor patient prognosis^112^. Mechanistically, the transcription factor NF-κB upregulates the expression of NKILA, which in turn binds to NF-κB/IκB, and directly masks the phosphorylation motifs of IκB, resulting in the inhibition of IKK-induced IκB phosphorylation, and NF-κB activation, forming a negative regulatory loop (Fig. 3b). This regulatory mechanism is promoting tumorigenesis by inhibiting apoptosis and increasing invasion. More recently, NKILA has also been shown to regulate T-cells activation by inhibiting NF-κB activity, and ectopic expression of NKILA in tumor-specific CTLs and T_H_1 cells correlated with apoptosis and shorter patient overall survival^113^.

#### NEAT1

The Homo sapiens nuclear paraspeckle assembly transcript 1 (NEAT1) sequence is a product of the NEAT1 gene, which is located on chromosome 11. NEAT1 has been identified as a p53 target gene that plays an important role in the formation of paraspeckles, and is indispensable for cell-cycle arrest and apoptosis in response to genotoxic stress^114^. Therefore, it has a key function in suppressing neoplastic transformation and cancer onset. Indeed, in a pancreatic cancer mouse model, NEAT1 deficiency increased transformation and contributed to the development of premalignant pancreatic intraepithelial neoplasia and cystic lesions through global changes in gene expression^115^. Moreover, NEAT1 expression is downregulated in several cancers, while increased NEAT1 levels are correlated with better overall survival in colorectal cancer patients. In addition, elevated NEAT1 levels have been associated with enhanced apoptosis after DNA damage by irradiation of chronic lymphocytic leukemia cells^116,117^. However, NEAT1 role is controversial since several studies also characterized its oncogenic activity. As matter of fact, during tumorigenesis NEAT1 levels are increased, and high levels of NEAT1 were associated with worse prognosis in gastric adenocarcinoma and laryngeal SCC^118,119^. Accordingly, modulation of NEAT1 expression promotes cell survival and/or proliferation of human cancer cell lines^120^, and NEAT1 acts as an oncogene in a mouse model of skin carcinogenesis^114^. At a molecular level, one possible mechanism by which NEAT1 exerts its oncogenic role, at least in ovarian cancer, is the interaction with the tumor suppressor miRNA miR-34a-5p, acting as a sponge and negatively regulating miR-34a expression.

Overall, NEAT1 can elicit a context-specific function in either promoting or suppressing neoplastic transformation.

### lncRNAs with anti-apoptotic functions

#### CCAT

Colon cancer-associated transcript (CCAT) is located within the 8q.24.21 genomic region that is frequently amplified in colorectal cancer (CRC)^121,122^. Expression profile studies highlight that CCAT1 and CCAT2 genes are frequently overexpressed in CRC, and their expression is associated with shorter progression-free and overall survival.

**Fig. 4:**
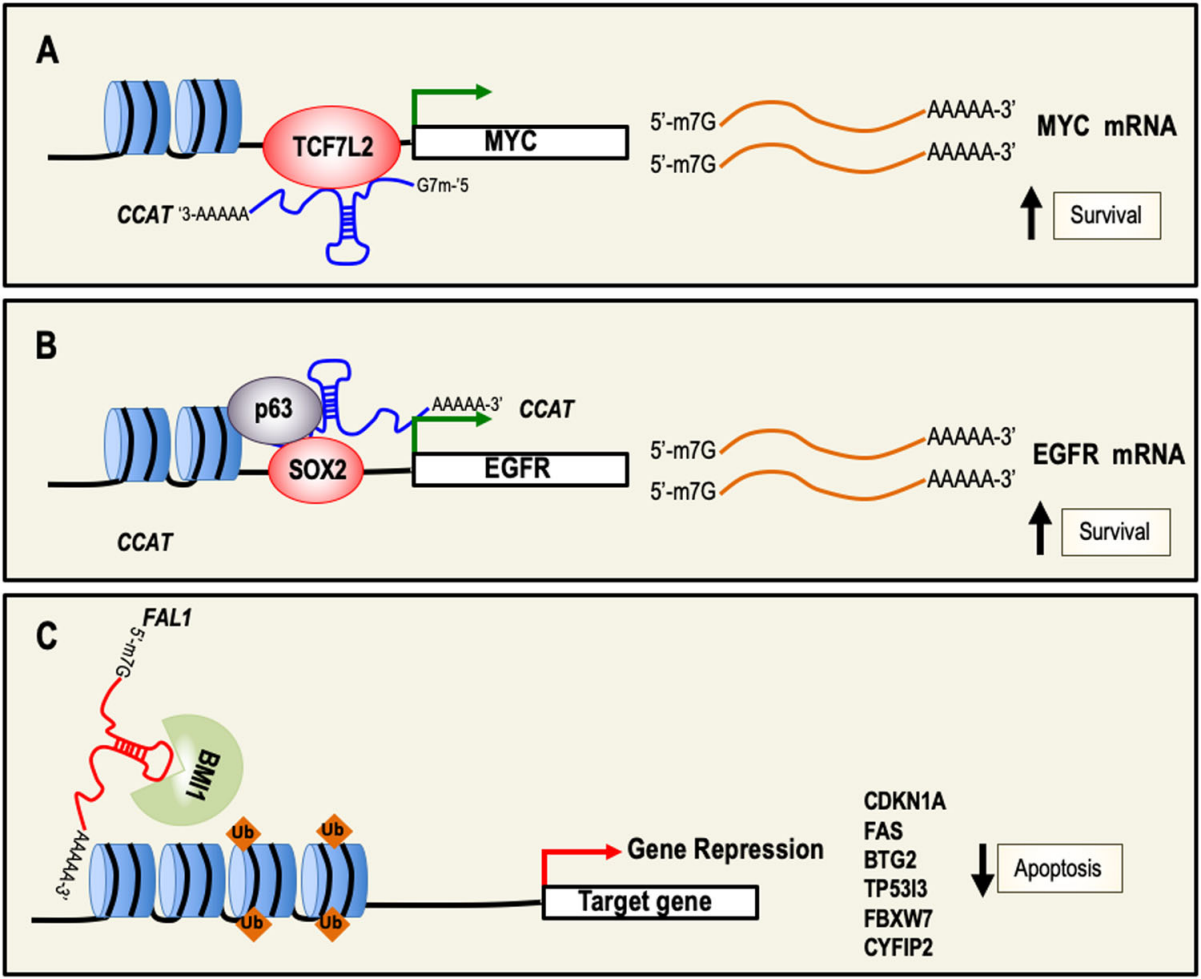
lncRNAs with an anti-apoptotic activity and relative molecular mechanisms. **a** CCAT is an oncogene that protects from cell death by up-regulating the expression of MYC. **b** p63, SOX2, and CCAT form a trimeric complex, which in turn binds the promoter region of EGFR and positively upregulates its expression. This results in sustaining cell survival. **c** FAL1 associates with the epigenetic repressor BMI1 and regulates its stability in order to modulate the transcription of a number of genes involved in cell death. CCAT colon cancer-associated transcript, MYC Myc proto-oncogene, SOX2 sex determining region box-2, EGFR epithelial growth factor receptor, FAL1 focally amplified LncRNA on chromosome 1, BMI1 polycomb complex protein BMI1.

The oncogenic function of CCAT is the result of the activation of genes involved in cell proliferation and in the inhibition of apoptosis. Indeed, CCAT regulates the overexpression of MYC, possibly through its physical interaction with TCF7L2, leading to genomic instability and promoting cell growth (Fig. 4a). In addition, a second possible molecular regulatory mechanism has been observed in SCC; in this context, CCAT regulates transcription of genes involved in cell proliferation and survival by a physical interaction with both transcription factors p63 and SOX2^123^. (Fig. 4b). Finally, overexpression of CCAT has been observed in other types of cancer, including gastric, breast, and lung cancer^124^.

#### FAL1

The gene encodes for focally amplified LncRNA on chromosome 1 (FAL1), and is localized at the chromosomal region 1q21.2. FAL1 functions as an oncogene in several cancers, and is upregulated in ovarian, thyroid cancer and NSCLC^125,126^. In addition, FAL1 expression correlates with some clinical and pathological characteristics of NSCLC and ovarian cancer patients. Both in vitro and in vivo studies demonstrated that the biological function of FAL1 is to regulate cell cycle, cell death, migration, and invasion^127^. Mechanistically, FAL1 interacts with the epigenetic repressor BMI1 and modulates the transcription of a number of genes involved in cell-cycle arrest and apoptosis, such as CDKN1A, FAS, BTG2, TP53I3, FBXW7, and CYFIP2^128^ (Fig. 4c).

#### PVT1

The human PVT1 gene is located in the human chromosome 8q24 region close to the well-established oncogene MYC^129^. Interestingly, the amplification of 8q24 is a frequent event in a vast variety of cancers including CRC, where it is also associated with clinical findings of decreased overall survival^130,131^. PVT1 functions as an oncogene by inhibiting the apoptosis of tumor cells, promoting cell proliferation, and affecting tumor invasion and metastasis generation. However, a detailed molecular mechanism underlying its anti-apoptotic activity is still missing. Recently, a possible mechanism by which PVT1 inhibits cell death has been described in nasopharyngeal carcinomas. In particular, PVT1 downregulates the expression of cleaved caspase-9, caspase-7, and PARP, inhibiting apoptosis and also promoting radiation resistance^132,133^.

### Circular RNAs

Circular RNAs (circRNAs) were identified more than 20 years ago, and for many years have just been considered as secondary products of aberrant splicing processes^134^. Only recently, with the advent of next-generation sequencing, a large number of circRNAs has been identified, several of them showing high and stable expression. Nearly 10% of genes transcribed in cells can produce circRNAs.

CircRNAs are transcribed by RNA polymerase II (Pol II), and they are generated by alternative splicing of pre-mRNA, in which the 5′ and the 3′ ends are joined together by a covalent bond, forming a single-strand continuous loop structure in a process known as “backsplicing”^135^. Moreover, several molecular mechanisms of action have been proposed to characterize their role in different biological and pathological processes. In particular, circRNAs are likely to function as miRNA sponges, to enhance the transcription of their parental genes or acting as decoys or scaffolds for proteins^136,137^.

The research on circRNAs is still in his infancy and their specific functions in several physiological processes and in the pathogenesis of human diseases is still largely unknown. However, recently high-throughput sequencing studies on clinical tumor samples showed a deregulation of circRNAs expression in tumors^138,139^. In particular, their downregulation has been described in proliferative cells across different tumor types, indicating that some circRNAs may have tumor suppressive roles. Global transcriptome analysis on 144 samples of localized prostate cancer, identifying 76,311 circRNAs, showed a correlation of circRNAs expression with tumor aggressivity, suggesting that 171 circRNAs are essential to prostate cancer cell proliferation^140^.

Overall, studies on clinical samples together with in vitro experiments showed that circRNAs play a role in several hallmarks of cancer having either tumor suppressive or oncogenic functions^141–143^. To our knowledge, no circRNAs from the literature have a clear and defined role in cancer biology proven by in vitro or in vivo experiments or by clinical data. However, we have decided to describe one of the more studied circRNAs that has a role in cell death regulation.

#### circFOXO3

circFOXO3 is a circular transcript of 1435 nucleotides derived from the tumor suppressor gene FOXO3^144^. Several expression profile studies indicated that circFOXO3 is downregulated in several tumors such as breast and NSCLC, suggesting a possible role as a tumor suppressor gene^145,146^. Both gain and loss of function experiments showed that circFOXO3 function is associated with the induction of apoptosis and with the inhibition of cell-cycle progression and angiogenesis. At a molecular level, ectopic expression of circFOXO3 decreases the interaction between FOXO3 and MDM2, releasing FOXO3 from MDM2-dependent degradation, therefore increasing FOXO3 activity, promoting PUMA expression, and enhancing cell death^147^.

### Perspectives and conclusions

During the last decades, we have witnessed considerable developments in understanding the role of the regulatory ncRNAs in the regulation of physiological processes as well as in the pathogenesis of several diseases. Deregulation of ncRNAs expression has been largely documented during the initiation, progression, and dissemination of cancer, and preclinical studies have shown that they can be used as diagnostic and prognostic biomarkers. More importantly, it has been documented that ncRNAs can be released into the extracellular space and detected in body fluids (blood or urine) as circulating ncRNAs, potentially showing their application in liquid biopsies^148^. The clinical application of ncRNAs is already under evaluation in ongoing clinical trials trying to describe their role as biomarkers for patient survival, metastasis development prediction, or therapy response (Table 2).

**Table 2:** Current clinical trials involving ncRNAs.

ncRNA	Study title	Condition or disease	Interventions	Primary purpose	Clinical trails identifier	Status
miR-16	MesomiR 1: A Phase I Study of TargomiRs as 2nd or 3rd Line Treatment for Patients with Recurrent MPM and NSCLC	Mesothelioma Non-small cell lung cancer	Drug: TargomiRs	Treatment	NCT02369198	Completed
miR-34	Critical Role of MicroRNA-34a and MicroRNA-194 in Acute Myeloid Leukemia With CEBPA Mutations	Leukemia	Genetic: RNA analysis	Not Reported	NCT01057199	Completed
miR-29	Phase 1: safety, tolerability and pharmacokinetic study of MRG-201 in healthy volunteers	Healthy volunteers	Drug: MRG-201	Treatment	NCT02603224	Completed
miR-155	The Potential Role Of microRNA-155 And Telomerase Reverse Transcriptase In Diagnosis Of Non-Muscle Invasive Bladder Cancer And Their Pathological Correlation	Bladder cancer	Diagnostic test	Diagnostic	NCT03591367	Completed
miR-221	Clinical Significance of Hepatic and Circulating microRNAs miR-221 and miR-222 in Hepatocellular Carcinoma	Hepatocellular carcinoma	Analysis of microRNA expression	Not Reported	NCT02928627	Unknown
miRNA/lncRNA	Noncoding RNA in the exosome of the epithelial ovarian cancer	Ovarian cancer	Sequencing miRNA/lncRNA		NCT03738319	Recruiting
miRNA	Circulating miRNAs as biomarkers of hormone sensitivity in breast cancer	Breast cancer	Drugs: tamoxifen, letrozole, anastrozole, exemestane	Treatment	NCT01612871	Completed
miRNA	Circulating microRNA as disease markers in pediatric cancer	Leukemia lymphoma	Not reported	Observational	NCT01541800	Recruiting

Source: ClinicalTrials.gov.

As from above, regulatory ncRNAs play a key role in driving or preventing the process of tumorigenesis and preclinical studies indicate that modulation of their expression by mimetic or inhibitory oligonucleotides might be a novel therapeutic approach for cancer therapy. Indeed, a limited number of clinical trials using drugs based on ncRNAs, especially on microRNAs, are evaluating clinical safety, tolerability, and efficacy of this approach (Table 2). Although therapeutic applications are still at an early phase, a new chapter in the pharmacological treatment of human diseases has started. The recent approval of the RNA-targeting oligonucleotides drug Spinraza for the treatment of spinal muscular atrophy by FDA^149^ makes reasonable to believe that more ncRNAs-based drugs will be soon available on the patient’s bedside.
